# Evaluation of a New Mordant Based Haematoxylin Dye (Haematoxylin X) for Use in Clinical Pathology

**DOI:** 10.3389/bjbs.2023.11591

**Published:** 2023-09-25

**Authors:** J. A. Gabriel, C. D’Amico, U. Kosgodage, J. Satoc, N. Haine, S. Willis, G. E. Orchard

**Affiliations:** ^1^ St. John’s Dermatopathology, Tissue Sciences, Synnovis Analytics, St. Thomas’ Hospital, London, United Kingdom; ^2^ CellPath Ltd, Powys, United Kingdom

**Keywords:** Haematoxylin X, Mohs micrographic surgery, routine staining, haematoxylin, paraffin processed and frozen tissue

## Abstract

Recently, St John’s Dermatopathology Laboratory and CellPath Ltd have developed a new patented haematoxylin dye (Haematoxylin X) that utilises a chromium-based mordant (Chromium Sulphate). In this study, the performance of this new haematoxylin (Haematoxylin X) was compared against some commonly utilised alum-based haematoxylins (Carazzi’s, Harris’ and Mayer’s) when used as a part of formalin-fixed paraffin embedded (FFPE) tissue, special stains, immunohistochemical counterstaining and frozen section (Mohs procedure) staining procedures. FFPE sections of different tissue types and frozen skin tissues were sectioned and stained with each haematoxylin subtype to allow for a direct comparison of staining quality. The slides were independently evaluated microscopically by two assessors. A combined score was generated to determine the sensitivity (defined as the intensity of haematoxylin staining being too weak or too strong and the colour of the haematoxylin staining not being blue/black) and specificity (defined as the presence of haematoxylin background staining, uneven staining, and staining deposits) for each of the four haematoxylin subtypes. The scoring criteria were based on the UKNEQAS Cellular pathology techniques assessment criteria. In FFPE tissue, the results for specificity identified Harris haematoxylin scoring the highest (91.2%) followed by Haematoxylin X (88.0%) and Mayer’s (87.0%). The sensitivity scores again identified Harris haematoxylin as scoring the highest (95.1%) followed by Haematoxylin X (90.0%) and Mayer’s (88.0%). In frozen tissue, the results for specificity identified Haematoxylin X as scoring the highest (85.5%) followed by Carazzi’s (80.7%) and Harris’ (77.4%). The sensitivity scores again identified Haematoxylin X as scoring the highest (86.8%) followed by Carazzi’s (82.0%) and Harris’ (81.0%). The results achieved with all four haematoxylins showed a high degree of comparability, with Harris’ haematoxylin scoring high scores overall compared to the other four when assessing FFPE sections. This may have been due to familiarity with the use of Harris’ haematoxylin in-house. There was also evidence of more pronounced staining of extracellular mucin proteins with Haematoxylin X compared to the other alum haematoxylins that were assessed. Haematoxylin X scored highest when used in frozen section staining. In addition, Haematoxylin X has a potential applications for use in IHC and special stains procedures as a counterstain.

## Introduction

The haematoxylin dye remains the gold standard for microscopic nuclear visualisation of cellular and tissue components in Histopathology [1]. When used in combination with eosin dye, it remains the staple in the interpretation of pathological changes of tissue sections under microscopic evaluation. However, whilst most diagnostic tools have advanced in the last decades, the haematoxylin dyes have remained relatively unchanged since the 1980s. Recently, St John’s Dermatopathology Laboratory and CellPath Ltd developed a new patented haematoxylin dye (Haematoxylin X) that utilises a chromium-based mordant (Chromium Sulphate) [2]. In this study, the performance of this new haematoxylin (Haematoxylin X) was compared against some commonly utilised alum-based haematoxylins (Carazzi’s, Harris’ and Mayer’s) when used as a part of formalin-fixed paraffin embedded (FFPE) tissue, special stains, immunohistochemical counterstaining and frozen section (Mohs procedure) staining procedures.

The haematoxylin dye is extracted from the bark of the logwood tree *Haematoxylum campechianum* [2]. The logwood tree genus *Haematoxylum* is derived from the Greek word haima: blood and xylon: wood, which is in reference to the dark-red colour of the logwood [3]. The species name *campechianum* is in reference to its city of origin, which is Campeche, located on the Yucatan peninsula of Mexico [3].

The haematoxylin dye, which is weakly anionic in nature, once bound to a mordant becomes basic and positively charged [4]. This results in the dye binding to substances which are acidic in nature resulting in its ability to bind to DNA/RNA, which are basophilic and negatively charged due to the phosphate backbone in their structure [4]. The negatively charged backbone binds with the basic dye resulting in the formation of salts [4]. However, the haematoxylin dye itself does not stain the cellular components, but the oxidised form haematin, which achieves the distinct blue/black colour [5]. The conversion of haematoxylin to haematin is achieved by one of two methods;

1) Natural oxidation (ripening), which achieves oxidation with exposure to air and light. This is the slower of the two options and can take up to 4–10 weeks [3].
2) Chemical oxidations achieve conversion by using a chemical agent (e.g., Sodium Iodate or Mercuric Oxide). It is the faster of the two methods and can achieve results with immediate effect [3].

Haematin is anionic in nature, resulting in a reduced ability to bind to tissue, with nuclear staining only achieved with the use of a mordant [4]. The mordants utilised are usually metal cations such as Chromium, Iron, Aluminium, Molybdenum, Lead and Tungsten [4]. These heavy metal salts or hydroxides provide a valency of two or three, resulting in a net positive charge to the complex formed by replacing a hydrogen atom from the haematoxylin dye, allowing the anionic dye to bind to cellular components [4]. There are a broad range of mordants utilised, which can impact the tissue components stained and the colour of staining.

There has been little advancement in the haematoxylin dye chemistry since the 1980s. The lack of research into new methods or formulations (e.g., new mordants) can hinder any improvements in quality of performance and efficiency. The development of Haematoxylin X, which utilises a novel Chromium Sulphate mordant, is the first major change in haematoxylin composition in decades. In this study, we assessed the performance of this new haematoxylin (Haematoxylin X) against some commonly utilised alum-based haematoxylins (Carazzi’s, Mayers and Harris) when used as a part of formalin-fixed paraffin-embedded (FFPE) tissue sections, special stains, immunohistochemistry (IHC) counterstaining and frozen section (Mohs procedure) staining process. The selection of the three alum haematoxylins was based on the author’s previous publication, which assessed the staining quality of nine different haematoxylin subtypes [6]. The final three selected represented dyes that scored across the spectrum of well, moderately, and poorly as determined by the criteria set out in Table 1.

**TABLE 1:** Factors that were used to assess the sensitivity and specificity of all haematoxylin subtypes.

Sensitivity factors	Specificity factors
-Haematoxylin intensity too strong	-Haematoxylin background staining
-Haematoxylin Intensity too weak	-Uneven staining
-Haematoxylin colour not purple/blue	-Stain deposit present
-Clarity of chromatin detail	-Non-specific staining of cells/tissue
-Crisp and clear demonstration of nucleoli	-Poor haematoxylin to eosin balance

## Materials and Methods

As part of the FFPE tissue staining analysis, ten tissue types were selected, comprising of normal skin, fibrofatty tissue (lipoma), small bowel, liver, breast, prostate, uterus, keloid and lymph node material. For each tissue type, ten different tissue blocks were generated, with one section being cut on each to produce 100 slides. The samples were first fixed in 10% neutral buffered formalin (Genta Medical Ltd, York, United Kingdom, see Table 2) for 24 h before undergoing paraffin wax processing on a routine overnight protocol (shown in Table 3) set up on a Sakura VIP 6AI tissue processor (Sakura Finetek, Thatcham, United Kingdom). Following the embedding of each of the ten tissues into histological cassettes, the samples were sectioned at 4um and mounted on uncharged glass microscope slides (CellPath Ltd, Powys, United Kingdom, see Table 2). To ensure adequate adherence of the sections to the glass slides, the tissue sections were baked in a 68°C oven for 20 min before the staining process was initiated.

**TABLE 2:** Reagent and consumable list.

Product name	Volume	Supplier/Manufacturer	Product code	Dilution (if applicable)
Carazzi’s	1 L	CellPath Ltd	HST001	N/a
Harris Haematoxylin	5 L	Leica Microsystem Ltd	3801560BBE	N/a
Haematoxylin X	1 L	CellPath Ltd	TBC	N/a
Mayer’s	1 L	Solmedia Ltd	HST011	N/a
Eosin	5 L	Leica Microsystem Ltd	3801590BBE	For Frozen protocol: 0.125% (Mix 625 mL of eosin with 4375 mL of distilled water
Scott’s Tap Water	5 L	Leica Microsystem Ltd	3802901E	N/a
Hydrochloric acid (for acid alcohol)	2 L	VWR International Ltd	20255.29	For frozen protocol: 0.225% (Mix 12.50 mL of HCL with 5 L of IDA) For paraffin protocol: 1% (Mix 50 mL of HCL with 5 L of IDA99%)
32% Ammonia (for ammonia alcohol)	2 L	VWR International Ltd	21192.298	For paraffin protocol: 0.13% (Mix 6.5 mL of Ammonia with 4,993.5 mL of IDA99%)
Industrial Denatured Alcohol 99% (IDA 99%)	5 L	Genta Medical Ltd	I99050	N/a
10% neutral buffered formalin	5 L	Genta Medical Ltd	BFN050	N/a
Xylene	5 L	Genta Medical Ltd	XYL050	N/a
CV mount	250 mL	Leica Biosystem Ltd	14046430011	N/a
Super frost slide	N/a	VWR International Ltd	631-0108	N/a
CLARITEX SLIDE, PINKCOAT, 1.0–1.2 mm, 90°, GROUND	N/a	CellPath Ltd	MAF-0108-03A	N/a
Coverglass	N/a	Leica Biosystem Ltd	3800146G	N/a
Glacial Acetic Acid	1 L	VWR International Ltd	84528.29	N/a
Periodic Acid 50%	100 mL	VWR International Ltd	294604D	N/a
Schiff’s	500 mL	Merck Life science UK	1090330500	N/a
Alcian Blue	100 g	Merck Life Science UK	A5268-100G	N/a
Ponceau Xylidine	50 g	Merck Life Science UK	P2395-50G	N/a
Acid Fuchsin	100 g	Merck Life Science UK	A-3908-25G	N/a
Aniline Blue diammonium salt	50 g	Merck Life Science UK	415049-50G	N/a
BenchMark Ultra LCS	N/a	Roche Diagnostics Ltd	5424534001	N/a
EZ PREP SOLUTION 10X	N/a	Roche Diagnostics Ltd	5279771001	N/a

**TABLE 3:** Tissue processor protocol for routine overnight processing.

Reagent	Duration (Hr)	P/V	Temperature°C	Mix
100% Alcohol	3	P/V	37	Continued
100% Alcohol	0.5	P/V	37	Continued
100% Alcohol	0.5	P/V	37	Continued
100% Alcohol	0.5	P/V	37	Continued
100% Alcohol	0.5	P/V	37	Continued
100% Alcohol	0.5	P/V	37	Continued
100% Alcohol	0.5	P/V	37	Continued
Xylene	1	P/V	37	Continued
Xylene	1	P/V	37	Continued
Xylene	1.5	P/V	37	Continued
Paraffin Wax	1	P/V	65	Continued
Paraffin Wax	1	P/V	65	Continued
Paraffin Wax	1	P/V	65	Continued
Paraffin Wax	1	P/V	65	Continued

To ensure standardisation of the staining process, the staining was performed on an automated Leica XL autostainer (Leica Biosystems, Milton Keynes, United Kingdom). The staining protocol for each of the four haematoxylin dyes (Haematoxylin X, Harris’, Mayer’s and Carazzi’s) was individually optimised by increasing or decreasing the immersion times in haematoxylin and/or acid alcohol. The concentration and timing of the remaining reagent constituents of the H&E staining process (i.e., eosin, ammoniated alcohol, industrial denatured alcohol 99%, and xylene) remained unchanged for all four haematoxylin dyes. Before the staining process, each haematoxylin dye was filtered before use to remove any precipitate that may have formed. The optimal staining protocol for each of the four dyes was determined by the microscopic evaluation of the stained slides by two of the authors, who are approved UKNEQAS CPT scheme assessors (GEO and CD) to determine which variable (haematoxylin and/or acid alcohol immersion times) needed to be amended to produce the optimum quality of staining. The assessment was performed by comparing the staining against the assessment criteria set out in Table 1. The optimised protocol-stained slides were then independently reviewed by the UKNEQAS CPT team, who provided feedback on the staining quality. The finalised protocols for each haematoxylin subtype are shown in Table 4. The last step involved the dehydration and clearing of the slides in industrial denatured alcohol 99% (Genta Medical Ltd, York, United Kingdom, see Table 2) and xylene (Genta Medical Ltd, York, United Kingdom, see Table 2) before the application of a mountant (Leica CV mount, Leica Biosystems, Milton Keynes, United Kingdom, see Table 2) and a glass coverslip (Leica Biosystems, Milton Keynes, United Kingdom, see Table 2).

**TABLE 4:** H&E protocols times for the staining of FFPE tissue by each haematoxylin subtype.

Haematoxylin subtype	Xylene X3 (mins)	Industrial denatured alcohol 99% X3 (mins)	Wash in running water (mins)	Time in haematoxylin (mins)	Wash in running water (mins)	Time in acid alcohol (seconds)	Wash in running water (mins)	Time in ammoniated alcohol (mins)	Wash in running water (mins)	Time in Eosin (mins)	Wash in running water (sec)	Industrial denatured alcohol 99% X3 (mins)	Xylene X3 (mins)
Carazzi’s	2	2	2	8	1	3	1	1	1	4	40	2	1
Harris	2	2	2	6	1	3	1	1	1	4	40	2	1
Haematoxylin X	2	2	2	8	1	3	1	1	1	4	40	2	1
Mayer’s	2	2	2	8	1	3	1	1	1	4	40	2	1

As part of the fresh tissue staining analysis, 250 anonymised sample tissues were selected from patients who had undergone the Mohs procedure for the treatment of cutaneous malignancies. All the samples were sectioned at 15 µm thickness on a Leica CM 1950 cryostat (Leica Biosystems, Milton Keynes, United Kingdom) and picked up on a charged Super frost glass slides (VWR international, Leicestershire, England, see Table 2). Before the initiation of the staining process, all slides were baked on a hot plate set at 80°C for 2 min, followed by 2 min in industrial denatured alcohol 99% (Genta Medical Ltd, York, United Kingdom, see Table 2) and water for an additional 3 min. To allow for increased standardisation, all staining of the fresh samples was carried out on a Linistat Linistainer (Thermofisher Scientific, Paisley, United Kingdom). Each of the haematoxylins were individually optimised by staining debulk sections, by increasing or decreasing the immersion times in haematoxylin and/or acid alcohol. As with FFPE staining process, the concentration and timing of the remaining reagents constituents of the H&E staining process (i.e., eosin, Scott’s tap water, industrial denatured alcohol 99%, and xylene) remained unchanged for all four haematoxylin dyes. The optimal staining protocols were determined by the author’s GEO and JG (who are approved UKNEQAS CPT scheme assessors) by microscopic assessment of each stained slide to elucidate which variable was required to be amended (haematoxylin and/or acid alcohol immersion times) to improve the staining result. The slides were assessed against the criteria set out in Table 1. The optimised protocol-stained slides were then independently reviewed by the UKNEQAS CPT team, who provided feedback on the staining quality. The finalised staining protocol for fresh tissue samples for each of the haematoxylin dyes is shown in Table 5. Once the slides were stained, they were dehydrated and cleared. Finally, CV mountant (Leica Biosystems, Milton Keynes, United Kingdom, see Table 2) was applied and a glass coverslip (Leica Biosystems, Milton Keynes, United Kingdom, see Table 2) was placed on top.

**TABLE 5:** H&E protocols times for the staining of Fresh tissue by each haematoxylin subtype.

Haematoxylin subtype	Time in haematoxylin (seconds)	Wash in running water (seconds)	Time in acid alcohol (seconds)	Wash in running water (seconds)	Time in Scott’s tap water (seconds)	Wash in running water (seconds)	Time in Eosin (seconds)	Wash in water (seconds)	Industrial denatured alcohol 99% (seconds)
Carazzi’s	40	10	10	10	10	10	10	10	20
Harris	30	10	10	10	10	10	10	10	20
Haematoxylin X	50	10	10	10	10	10	10	10	20
Mayer’s	50	10	10	10	10	10	10	10	20

Upon completion of staining of all FFPE and fresh tissue cases with each of the haematoxylin dyes, the slides were assessed independently by two of the authors in a blind trial approach. The scoring of the slides was based upon a modified version of the United Kingdom External Quality Assurance Cellular Pathology Techniques (UKNEQAS CPT) Tissue Diagnostics and Mohs procedure assessment criteria [7, 8]. Each assessor allocated a score between 1 and 5 based on the modified UKNEQAS scoring criteria, with the assessment focusing on the quality of haematoxylin staining. The criteria utilised in this assessment are highlighted in Table 1.

The results assigned by each judge for the specificity and sensitivity of each slide were then combined to generate an overall score for each slide out of 10. These results were then added together and divided by 100 to calculate the mean. The mean was then divided by 10 and multiplied by 100 to generate sensitivity and specificity scores as a percentage for each haematoxylin dye. These sensitivity and specificity scores generated were critically evaluated to determine the staining quality of Haematoxylin X against the other commonly used haematoxylins.

To perform the special stains, initially, the FFPE blocks were sectioned at 4 µm thickness on a Leica Histocore biocut rotary microtome (Leica Biosystems, Milton Keynes, United Kingdom) and picked up on a charged Super frost glass slides (VWR international, Leicestershire, England, see Table 2). The slides were then baked on a hotplate set at 80°C for 15 min. The slides were then deparaffinised by running through three changes of xylene (Genta Medical Ltd, York, United Kingdom, see Table 2) and IDA99% (Genta Medical Ltd, York, United Kingdom, see Table 2), then into running tap water.

To perform the periodic acid Schiff stain the slides were treated with 1% periodic acid (VWR international, Leicestershire, England, see Table 2) for 10 min and then rinsed in running tap water. The slides were then treated in Schiff’s reagent (Merck Life Science, Dorset, United Kingdom, see Table 2) for 20 min and then rinsed in running tap water for 10 min. Next, the sections were counterstained with Haematoxylin X for 2 min, rinsed and then differentiated in 1% acid alcohol for 5 s. The rinsed slides were then treated with ammoniated alcohol for 1 min and then rinsed in running tap water. Lastly, the slide was dehydrated rapidly in IDA 99% (Genta Medical Ltd, York, United Kingdom, see Table 2), cleared in xylene (Genta Medical Ltd, York, United Kingdom, see Table 2), and mounted with CV mount (Leica Biosystems, Milton Keynes, United Kingdom, see Table 2) and a cover glass (Leica Biosystems, Milton Keynes, United Kingdom, see Table 2).

To perform the Alcian blue stain, the sections were first stained with filtered Alcian blue solution (Merck Life Science, Dorset, United Kingdom, see Table 2) for 10 min. Next, the sections were rinsed with running tap water and then counterstained with Haematoxylin X for 5 min, rinsed and then differentiated in 1% acid alcohol for 5 s. The rinsed slides were then treated with ammoniated alcohol for 1 min and then rinsed in running tap water. Lastly, the slide was dehydrated rapidly in IDA 99% (Genta Medical Ltd, York, United Kingdom, see Table 2), cleared in xylene (Genta Medical Ltd, York, United Kingdom, see Table 2), and mounted with CV mount (Leica Biosystems, Milton Keynes, United Kingdom, see Table 2) and a cover glass (Leica Biosystems, Milton Keynes, United Kingdom, see Table 2).

To perform the Masson trichrome stain, the slide was stained with Haematoxylin X for 20 min and then washed in running tap water. The sections were then differentiated in 1% acid alcohol for 10 s and then blued in running tap water. Next, the sections were stained with the red cytoplasmic stain (two parts 1% Ponceau de Xylidine (Ponceau 2R) in 1% acetic acid and one part 1% acid fuchsin in 1% acetic acid) for 10 min and then washed in running tap water. The sections were differentiated in 1% phosphomolybdic acid until the collagen was decolourised and the muscle fibres, red blood cells and fibrin remained red. Once the slide was rinsed in distilled water, the section was stained with 0.5% aniline blue in 1% acetic acid for 1 min and then rinsed in 1% acetic acid (VWR international, Leicestershire, England, see Table 2). Lastly, the slide was blot dried, dehydrated rapidly in IDA 99% (Genta Medical Ltd, York, United Kingdom, see Table 2), cleared in xylene (Genta Medical Ltd, York, United Kingdom, see Table 2), and mounted with CV mount (Leica Biosystems, Milton Keynes, United Kingdom, see Table 2) and a cover glass (Leica Biosystems, Milton Keynes, United Kingdom, see Table 2).

To perform manual counterstaining in immunohistochemical staining, the slides were first allowed to stain in the Roche Ventana Benchmark Ultra (Roche Diagnostics Ltd, Burgess Hill, United Kingdom) without the selection of counterstaining. The slides were then removed before being washed with EZ prep solution (Roche Diagnostics Ltd, Burgess Hill, United Kingdom) and running water to remove any excess LCS solution (Roche Diagnostics Ltd, Burgess Hill, United Kingdom). The slides were then placed in filtered Haematoxylin X and allowed to stain for 3 min. The slides were then washed in running water and differentiated in acid alcohol for 3 s. Next, the slides were washed in running water and then blued in ammoniated alcohol for 30 s. Lastly, the slide was dehydrated rapidly in IDA 99% (Genta Medical Ltd, York, United Kingdom, see Table 2), cleared in xylene (Genta Medical Ltd, York, United Kingdom, see Table 2), and mounted with CV mount (Leica Biosystems, Milton Keynes, United Kingdom, see Table 2) and a cover glass (Leica Biosystems, Milton Keynes, United Kingdom, see Table 2).

## Results

**FIGURE 1:**
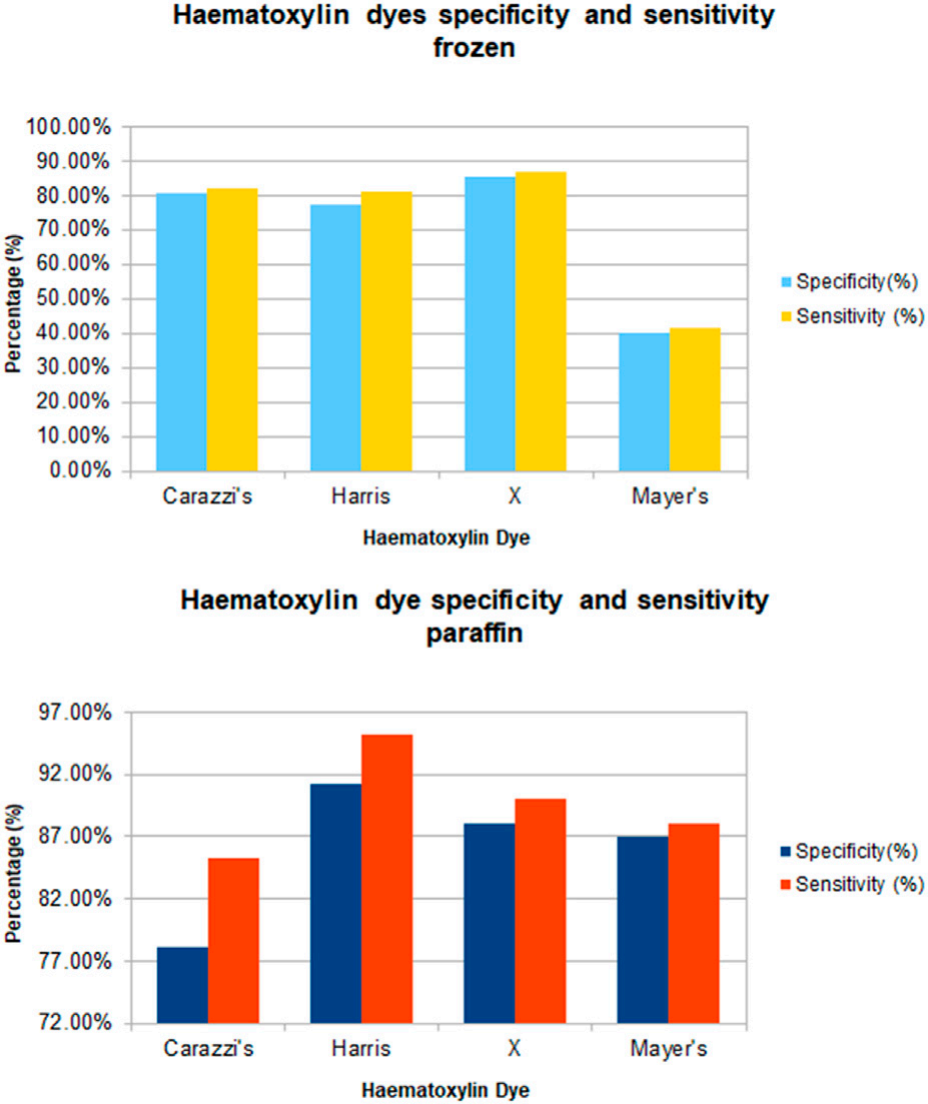
Graph of sensitivity and specificity of all haematoxylin stain subtype for frozen and paraffin sections.

All the slides evaluated, which were stained with each of the four haematoxylin dyes, stained as expected with varying degrees of nuclear staining intensity. The specificity and sensitivity results generated for each of the four haematoxylins as based on the UKNEQAS criteria set out in Table 1 are shown in Table 6. Figure 1 shows the specificity and sensitivity in a graphical format.

**TABLE 6:** Breakdown of specificity and sensitivity result for each haematoxylin subtype for frozen and paraffin sections.

Frozen			
Haematoxylin subtype	Mordant	Specificity (%)	Sensitivity (%)
Carazzi’s	Potassium Alum	80.7	82.0%
Harris	Potassium Alum	77.4	81.0%
X	Chromium Sulphate	85.5	86.8%
Mayer’s	Potassium Alum	40.0	41.4%
**Paraffin**			
**Haematoxylin Subtype**	**Mordant**	**Specificity (%)**	**Sensitivity (%)**
Carazzi’s	Potassium Alum	78.1%	85.2%
Harris	Potassium Alum	91.2%	95.1%
X	Chromium Sulphate	88.0%	90.0%
Mayer’s	Potassium Alum	87.0%	88.0%

**FIGURE 2:**
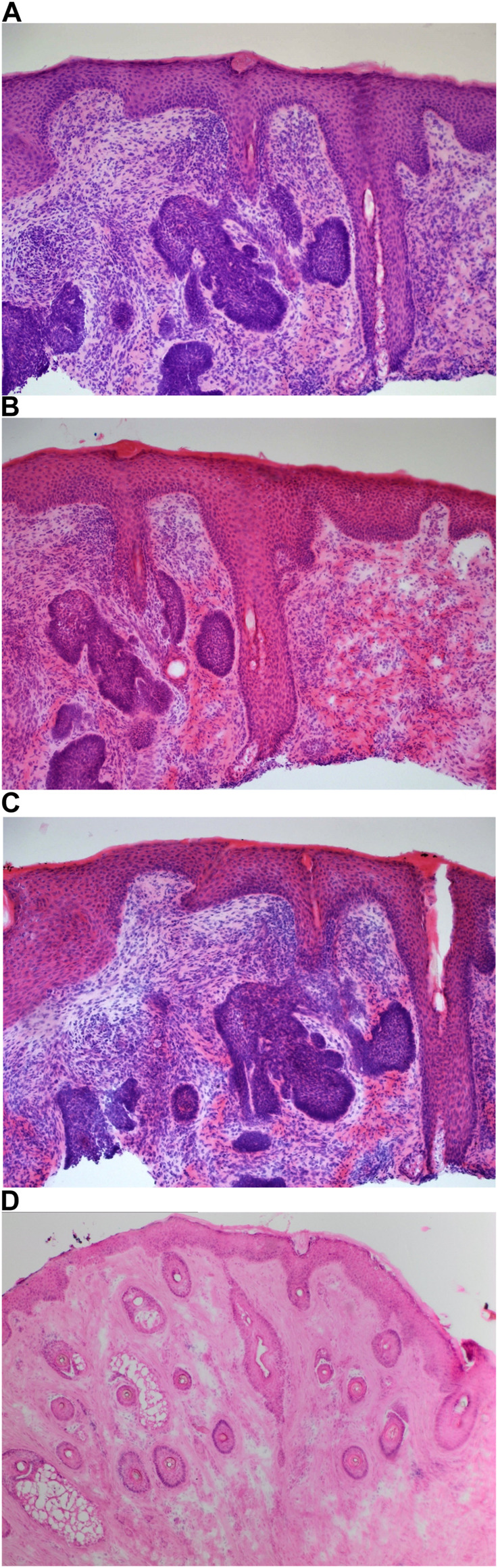
Example of frozen section of skin stained with different haematoxylins. **(A)** (×20 magnification) shows a photomicrograph of an H&E section stained with Carazzi’s haematoxylin. **(B)** (×20 magnification) shows a photomicrograph of an H&E section stained with Harris’ haematoxylin. **(C)** (×20 magnification) shows a photomicrograph of an H&E section stained with Haematoyxlin X. **(D)** (×10 magnification) shows a photomicrograph of an H&E section stained with Mayer’s haematoxylin.

**FIGURE 3:**
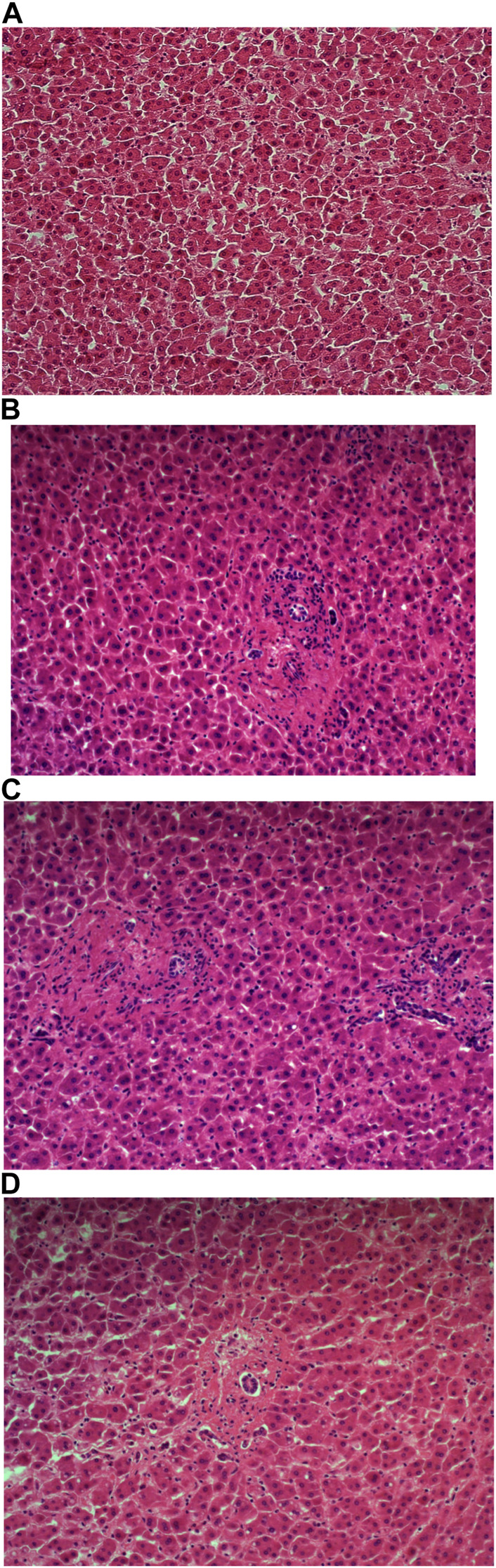
Example of paraffin section of liver stained with different haematoxylins. **(A)** (×20 magnification) shows a photomicrograph of an H&E section stained with Carazzi’s haematoxylin. **(B)** (×20 magnification) shows a photomicrograph of an H&E section stained with Harris’ haematoxylin. **(C)** (×20 magnification) shows a photomicrograph of an H&E section stained with Haematoyxlin X. **(D)** (×20 magnification) shows a photomicrograph of an H&E section stained with Mayer’s haematoxylin.

**FIGURE 4:**
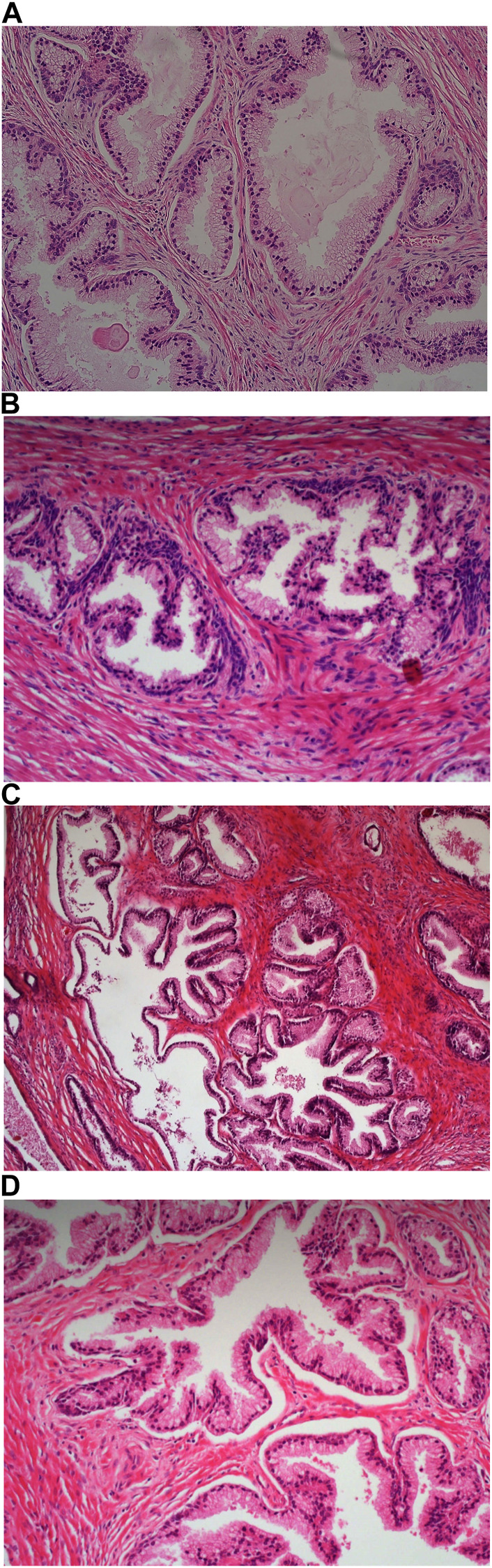
Example of paraffin section of prostate stained with different haematoxylins. **(A)** (×20 magnification) shows a photomicrograph of an H&E section stained with Carazzi’s haematoxylin. **(B)** (×20 magnification) shows a photomicrograph of an H&E section stained with Harris’ haematoxylin. **(C)** (×20 magnification) shows a photomicrograph of an H&E section stained with Haematoyxlin X. **(D)** (×20 magnification) shows a photomicrograph of an H&E section stained with Mayer’s haematoxylin.

**FIGURE 5:**
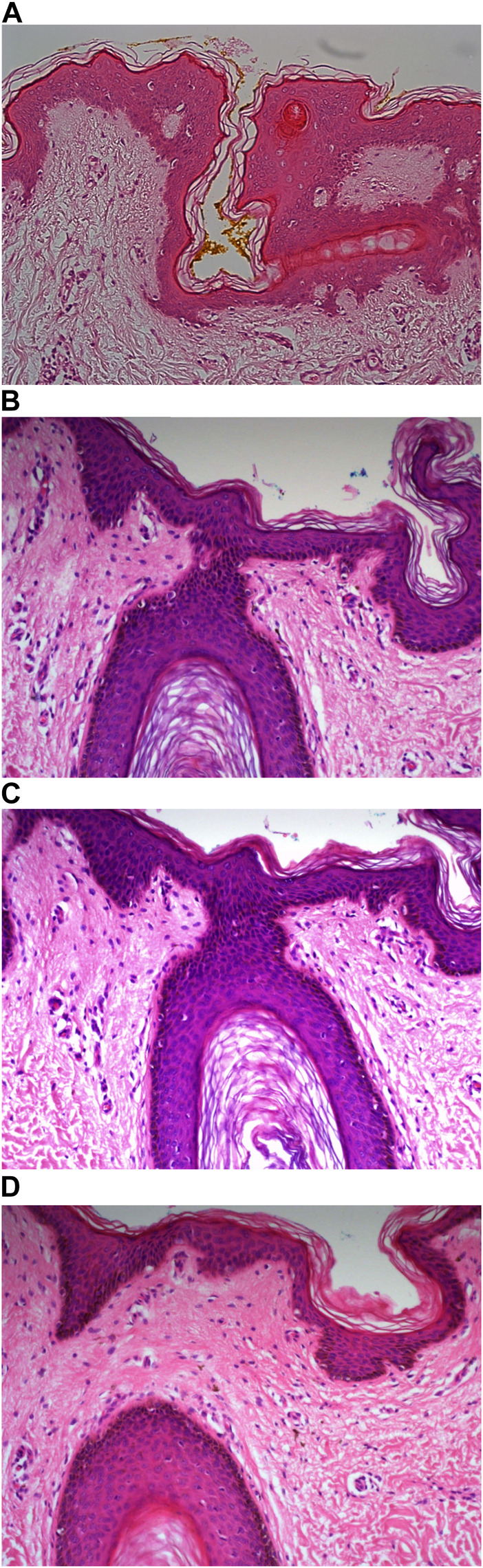
Example of paraffin section of skin stained with different haematoxylins. **(A)** (×20 magnification) shows a photomicrograph of an H&E section stained with Carazzi’s haematoxylin. **(B)** (×20 magnification) shows a photomicrograph of an H&E section stained with Harris’ haematoxylin. **(C)** (×20 magnification) shows a photomicrograph of an H&E section stained with Haematoyxlin X. **(D)** (×20 magnification) shows a photomicrograph of an H&E section stained with Mayer’s haematoxylin.

**FIGURE 6:**
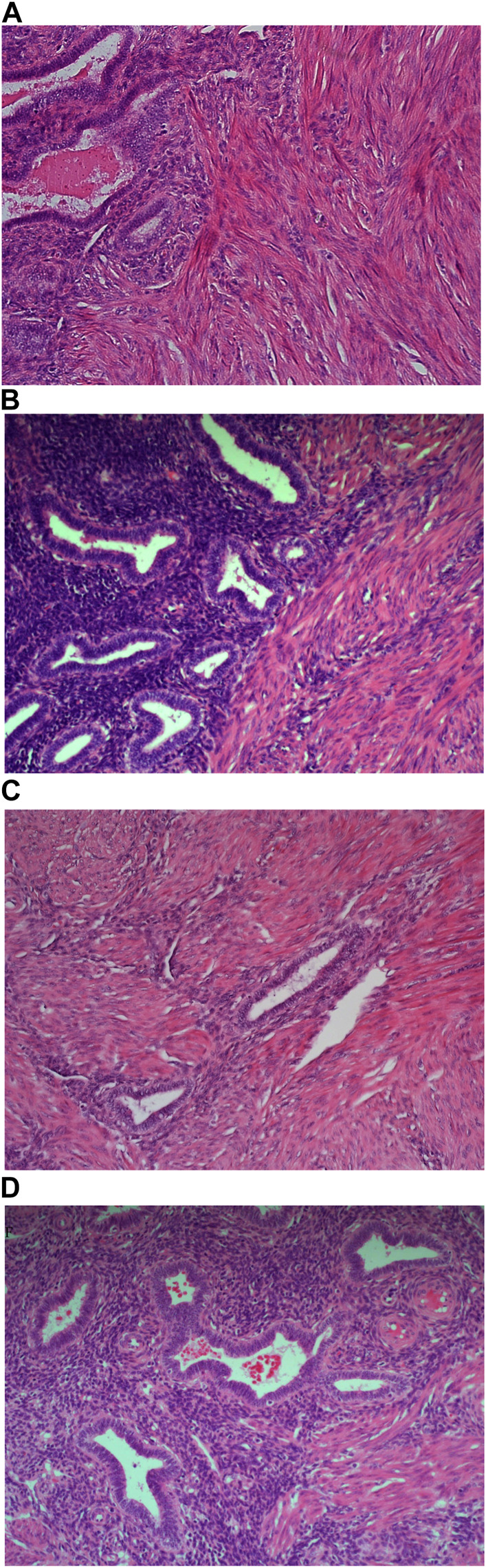
Example of paraffin section of Uterus stained with different haematoxylins. **(A)** (×20 magnification) shows a photomicrograph of an H&E section stained with Carazzi’s haematoxylin. **(B)** (×20 magnification) shows a photomicrograph of an H&E section stained with Harris’ haematoxylin. **(C)** (×20 magnification) shows a photomicrograph of an H&E section stained with Haematoyxlin X. **(D)** (×20 magnification) shows a photomicrograph of an H&E section stained with Mayer’s haematoxylin.

Carazzi’s haematoxylin-stained fresh tissue sections produced good quality staining with clear visualisation of the nucleoli and chromatin details. There was an acceptable contrast between the eosin and haematoxylin staining, minimal background and uneven staining patterns (see Figure 2A). Carazzi’s haematoxylin received the second highest score for specificity and sensitivity of 80.7% and 82.0%. In contrast, when used on FFPE tissue, Carazzi’s haematoxylin produced a paler nuclear staining. However, the chromatin and nucleoli detail was still visible. There was a poorer haematoxylin-to-eosin balance due to the eosin overpowering the weaker haematoxylin staining (see Figures 3A, 4A, 5A, 6A). Overall, Carazzi’s haematoxylin scored 78.1% and 85.2% for specificity and sensitivity, respectively.

Harris haematoxylin-stained fresh tissue produced satisfactory nuclear staining with an adequate interpretation of the chromatin detail and nucleoli. The intensity of haematoxylin staining was variable amongst the cohort of slides stained. This may be due to variation in the fixation process of the tissue. Factors relating to the specificity that was observed included uneven staining patterns, and due to the weaker haematoxylin, there were areas of more pronounced eosin staining (see Figure 2B). Harris’ haematoxylin scored 77.4% and 81.0% for specificity and sensitivity, respectively. Harris haematoxylin stained FFPE tissue produced a good level of nuclear staining with clear and crisp visualisation of the nucleoli and chromatin detail. There was also minimal background staining, uneven staining and staining deposits observed (see Figures 3B, 4B, 5B, 6B). Overall, Harris haematoxylin ranked first for specificity and sensitivity, with scores of 91.2% and 95.1%.

Haematoxylin X-stained fresh tissue produced good quality nuclear staining with clear and crisp nucleoli and chromatin detail visualisation. The intensity of haematoxylin could be variable at times. The vast majority of factors relating to sensitivity were adhered to with only minimal areas of uneven staining due to slightly weaker haematoxylin nuclear staining in places (see Figure 2C). Overall, Haematoxylin X ranked first for sensitivity and specificity, with a score of 86.8% and 85.5%. Haematoxylin X-stained FFPE sections produced optimal nuclear staining allowing for good chromatin and nucleoli detail visualisation. Haematoxylin X-stained sections did not have the presence of factors related to specificity, with only a minimal level of uneven staining observed (see Figures 3C, 4C, 5C, 6C). Haematoxylin X ranked second for specificity and sensitivity, with results of 88.0% and 90.0%.

Mayer’s haematoxylin-stained fresh tissue produced suboptimal nuclear staining with pale haematoxylin staining. As a result, the nucleoli and chromatin detail visualisation were not as easily achieved. Whilst Mayer’s haematoxylin did not produce background staining, there was the presence of uneven staining and increased eosin staining in places due to weaker intensity of haematoxylin staining (see Figure 2D). Mayer’s haematoxylin ranked last for specificity and sensitivity, with scores of 40.0% and 41.4%. Mayer’s haematoxylin-stained FFPE tissue sections resulted in good nuclear staining, which allowed for the visualisation of the nucleoli and chromatin detail. Most of the factors relating to specificity were not present, but there were some uneven staining patterns observed (see Figures 3D, 4D, 5D, 6D). Mayer’s haematoxylin ranked third overall, with specificity and sensitivity scores of 87.0% and 88.0%.

**FIGURE 7:**
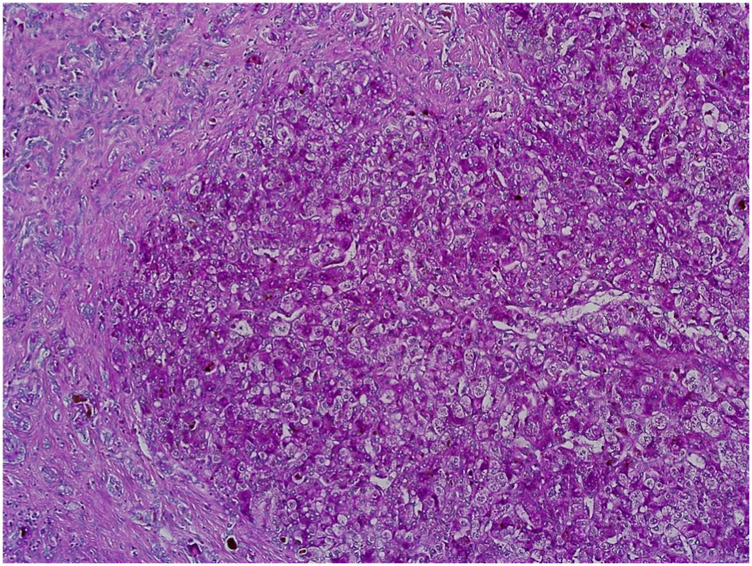
Example of paraffin section of Liver stained with periodic acid Schiff’s. (×20 magnification) shows a photomicrograph of a liver section stained with periodic acid Schiff’s stain.

**FIGURE 8:**
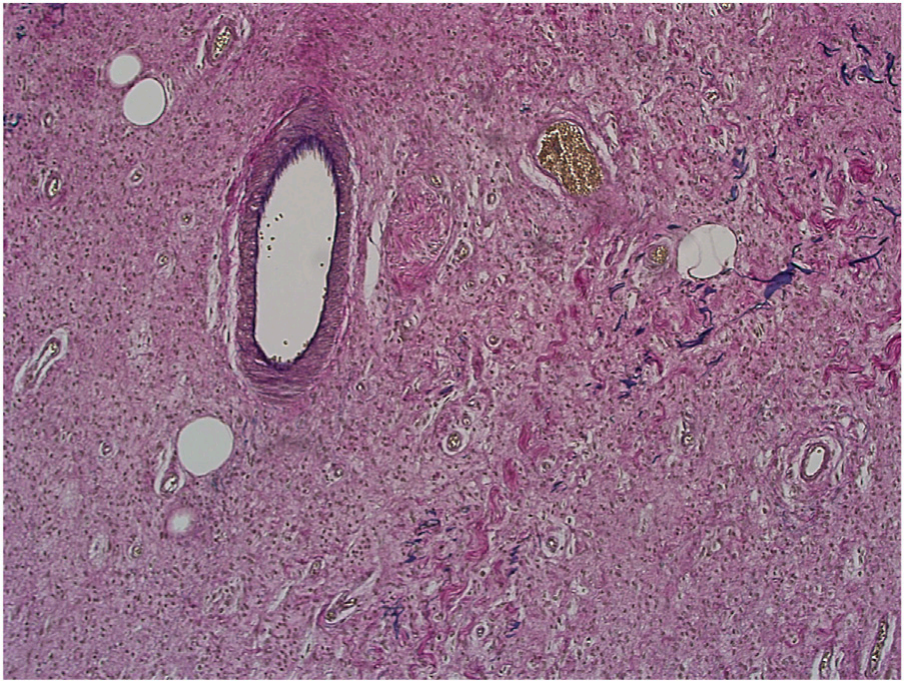
Example of paraffin section of skin stained with haematoxylin and Van Gieson’s stain. (×20 magnification) shows a photomicrograph of a skin section stained with haematoxylin and Van Gieson’s stain.

Evaluation of slides stained with PAS (see Figure 7), Alcian blue, HVG (see Figure 8) special stains and counterstained with Haematoxylin X produced good quality staining with an optimal level of nuclear staining with good contrast.

**FIGURE 9:**
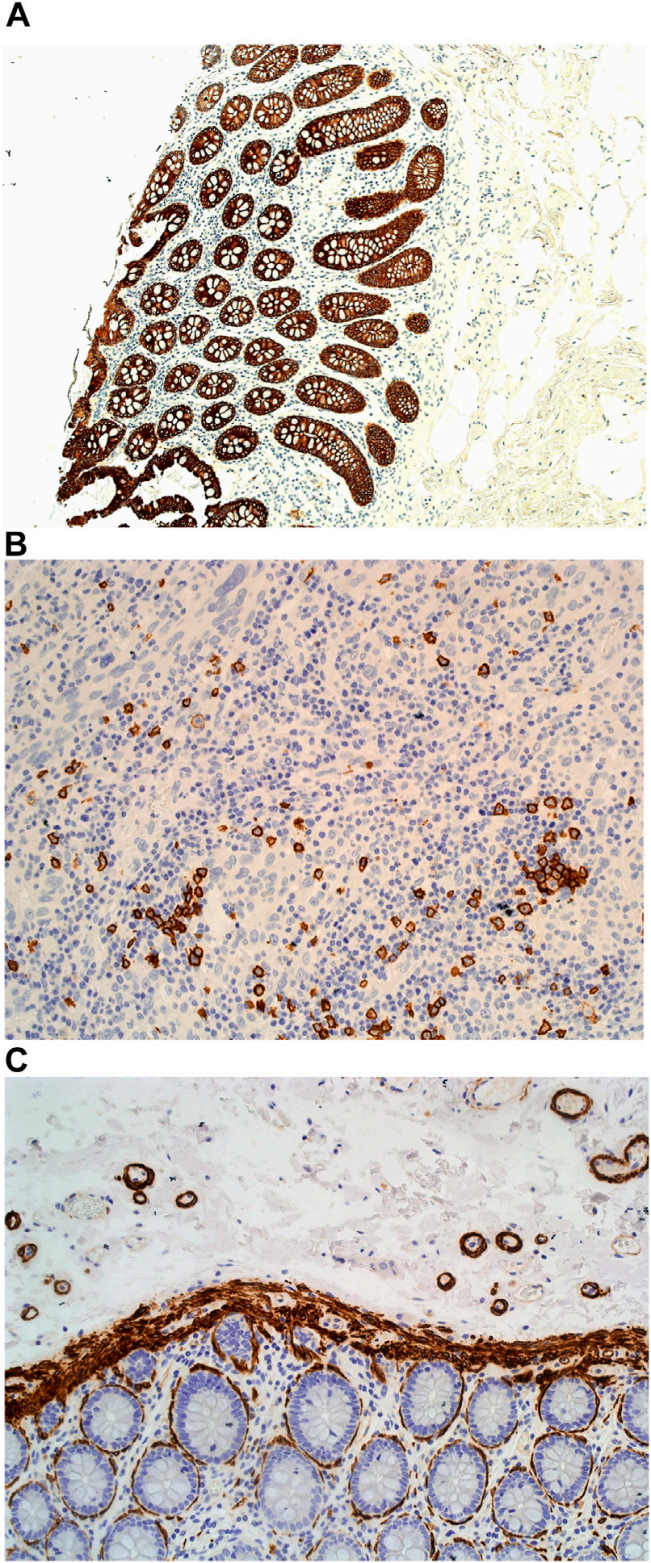
Example of paraffin sections stained with CD20 and MNF116. **(A)** (×20 magnification) shows a photomicrograph of a colon section stained with MNF116. **(B)** (×20 magnification) shows a photomicrograph of a tonsil section stained with CD20. **(C)** (×20 magnification) shows a photomicrograph of a colon section stained with Caldesmon.

However, when used in the Masson trichome staining, which encompasses acidic staining solutions, the Haematoxylin X nuclear staining was suboptimal and produced pale staining. When used as part of the immunohistochemistry staining process, Haematoxylin X produced clear and crisp nuclear staining. It produced good contrast, which allowed for a clear interpretation of the immunohistochemical staining (see Figures 9A–C).

## Discussion

The haematoxylin dye remains the most critical dye for the microscopic interpretation of nuclear detail, which is crucial in the diagnosis of many pathological disorders in histopathology. There has been little advancement in the composition of the haematoxylin dye in several decades. This lack of research and innovation can hinder any progress and improvements in the dye that could aid with producing an improved quality of nuclear staining, efficiency and cost-effectiveness of the dye. The development of a chromium sulphate mordant-based haematoxylin dye is the first major change in the haematoxylin composition since the 1980s. In the future, with the implementation of digital pathology systems and with artificial intelligence becoming a potential tool for the reporting of histological sections, the staining quality of H&E-stained slides becomes of greater importance. Whilst a pathologist may be able to adapt to staining heterogeneity and variability, AI systems are not, and this is the main challenge faced when utilising these new technologies [9–11]. This study highlights that Haematoxylin X produced good nuclear staining in both FFPE and fresh tissue.

The analysis of the tissue sections stained with each of the four different haematoxylin subtypes (Carazzi’s, Harris’, Haematoxylin X and Mayer’s) showed great comparability with the degree of nuclear staining. Haematoxylin X performed well in staining FFPE tissue and was only narrowly outperformed by Harris’ haematoxylin. This could be due to the fact that Harris’ haematoxylin is utilised as the routine dye, and as a result, the assessors are more familiar with this subtype. It was also noted that Haematoxylin X was efficacious in staining extracellular mucin, particularly in uterine tissue. While there was evidence of staining of mucins with Mayer’s and, to a lesser extent Harris’, Haematoxylin X produced the most prominent intensity of staining. This suggests that the new haematoxylin may have particular use in the morphological characterisation of mucin-producing tumours or associated pathologies, i.e., sebaceous carcinomas or adenocarcinomas.

Evaluation of fresh tissue sections stained with each of the haematoxylin subtypes again produced a comparable level of nuclear staining. Haematoxylin X produced the most optimal result when used as part of the frozen staining procedure when compared to the other alum-based haematoxylin dyes that were assessed. The clear and crisp nuclear staining allowed for ease of confirmation of basal cell carcinoma tumours from uninvolved epithelial cells. There was also clear visualisation of the chromatin and nucleoli detail which is essential in tumour characterisation. This supports the potential use of Haematoxylin X in rapid intraoperative procedures, which requires the performance of H&E staining on fresh tissue sections, i.e., Mohs micrographic surgery histological techniques. It was noted that Mayer’s haematoxylin produced sub-optimal staining quality as part of the Mohs frozen section staining in this study. It could be argued that the staining could have been improved if the immersion time was increased. However, the linistat linistainer is restricted to a maximum immersion time of 50 s. As a result, it was not feasible to increase the timing to improve the staining quality. Furthermore, intraoperative staining procedures such as Mohs require quick turn-around-times, as such, dyes which take longer would be less desirable than dyes which stain in a quicker time frame.

Haematoxylin X was observed to be suitable for use as part of both special staining (e.g., periodic acid Schiff’s and Alcian blue) and immunohistochemistry counterstaining, with clear and crisp nuclear staining observed with good contrast. However, as with other non-iron mordant-based haematoxylins, it was found that the chromium-sulphate based haematoxylin was also not suitable for use with stains that incorporated acid solutions such as trichrome stains.

An observation that was also noted with Haematoxylin X was that it did not produce oxides and precipitates when left exposed to the air at ambient temperatures, which was prominent with commonly utilised alum haematoxylins such as Harris’. However, it was noted that there were some minor precipitates present in the tissue sections when viewed under higher magnification (×60 or higher). This is believed to be due to the formation of chromium salts. The formation of these salts is eradicated by performing a filtration procedure during the manufacturing of the dye. This study was conducted with an initial small batch of the Haematoxylin X formulation made by CellPath Ltd. The dye is currently being upscaled for mass production and is still undergoing testing at the time of this publication.

The diagnosis and classification of most pathological disorders rely on the information gathered from the evaluation of H&E-stained sections, with the interpretation of haematoxylin-stained nuclear detail playing an essential role in determining morphological characteristics. This study shows that the new chromium sulphate mordant-based Haematoxylin X performed well in both FFPE and fresh tissue staining, highlighting the dye as a good candidate for use as part of routine diagnostic H&E staining.

## Summary Table

### What Is Known About This Subject?

• The haematoxylin dye remains the gold standard for microscopic nuclear visualisation of cellular and tissue components in Histopathology.
• However, whilst most diagnostic tools have advanced in the last decades, the haematoxylin dyes have remained relatively unchanged since the 1980s.

### What This Paper Adds

• The development of a chromium sulphate mordant-based haematoxylin dye (Haematoxylin X) constitutes a major change in the haematoxylin composition since the 1980s.
• Haematoxylin X produced good nuclear staining and may also have applications as a good counterstain for immunohistochemical staining and special stains.
• Haematoxylin X may also have a value in the morphological characterisation of mucin-producing tumours or associated pathologies.

## Data Availability

The original contributions presented in the study are included in the article/supplementary material, further inquiries can be directed to the corresponding author.
